# A novel method to identify high order gene-gene interactions in genome-wide association studies: Gene-based MDR

**DOI:** 10.1186/1471-2105-13-S9-S5

**Published:** 2012-06-11

**Authors:** Sohee Oh, Jaehoon Lee, Min-Seok Kwon, Bruce Weir, Kyooseob Ha, Taesung Park

**Affiliations:** 1 Department of Statistics, Seoul National University, Seoul, South Korea; 2 Interdisciplinary Program in Bioinformatics, Seoul National University, Seoul, South Korea; 3 Department of Biostatistics, University of Washington, Seattle, Washington, USA; 4 Department of Neuropsychiatry, Seoul National University College of Medicine, Seoul National University Bundang Hospital, Seongnam, Korea

## Abstract

**Background:**

Because common complex diseases are affected by multiple genes and environmental factors, it is essential to investigate gene-gene and/or gene-environment interactions to understand genetic architecture of complex diseases. After the great success of large scale genome-wide association (GWA) studies using the high density single nucleotide polymorphism (SNP) chips, the study of gene-gene interaction becomes a next challenge. Multifactor dimensionality reduction (MDR) analysis has been widely used for the gene-gene interaction analysis. In practice, however, it is not easy to perform high order gene-gene interaction analyses via MDR in genome-wide level because it requires exploring a huge search space and suffers from a computational burden due to high dimensionality.

**Results:**

We propose dimensional reduction analysis, Gene-MDR analysis for the fast and efficient high order gene-gene interaction analysis. The proposed Gene-MDR method is composed of two-step applications of MDR: within- and between-gene MDR analyses. First, within-gene MDR analysis summarizes each gene effect via MDR analysis by combining multiple SNPs from the same gene. Second, between-gene MDR analysis then performs interaction analysis using the summarized gene effects from within-gene MDR analysis. We apply the Gene-MDR method to bipolar disorder (BD) GWA data from Wellcome Trust Case Control Consortium (WTCCC). The results demonstrate that Gene-MDR is capable of detecting high order gene-gene interactions associated with BD.

**Conclusion:**

By reducing the dimension of genome-wide data from SNP level to gene level, Gene-MDR efficiently identifies high order gene-gene interactions. Therefore, Gene-MDR can provide the key to understand complex disease etiology.

## Background

With the development of high-throughput genotyping technologies, a genome-wide association (GWA) study has become a standard approach for testing association between a single nucleotide polymorphism (SNP) and a complex disease of interest such as diabetes, hypertension, schizophrenia, and bipolar disorder (BD) [1-4]. There have been many successful results from GWA studies, however, only a small number of genetic factors have passed the genome-wide significance and have been shown to explain only a small fraction of disease etiology due to ignoring relatedness between complex diseases and multiple genes and/or their interactions [5]. If a genetic factor functions primarily through a complex mechanism that involves multiple genes and environmental factors, the effect might be missed when the gene is examined in isolation without allowing for its potential interactions with other unknown factors [6]. Therefore, it is essential to investigate the gene-gene and/or gene-environment interactions in order to understand the etiology of common complex diseases thoroughly.

Several methods have been proposed to identify the gene-gene and/or gene-environment interactions. Among them, logistic regression is a most commonly used method to analyze the gene-gene interaction in genetic association studies [7-10]. However, when the SNPs are in linkage disequilibrium (LD), logistic regression encounters a multicollinearity problem. In addition, when there are empty or sparse cells, logistic regression has some possibilities of misleading inference. Thus, a large sample size is required for estimating logistic regression parameters to avoid sparseness problems for modeling high-order interactions.

To address the sparseness problem, the multifactor dimensionality reduction (MDR) method was proposed by Ritchie et al. [11]. The MDR method, as a non-parametric and model free method, has been widely used for detecting gene-gene interaction because it does not require any assumption of genetic mode of inheritance [11-14]. Besides, it has provided good performances for the small samples and in the presence of LD between genetic factors. MDR analysis identifies gene-gene interaction based on *k*-fold cross-validation (CV) to avoid overfitting problem and presents which genotype combinations are either high or low risk on disease of interest. Many research groups have investigated the extensions of MDR method [15-19]. For example, generalized MDR was proposed to handle quantitative traits and adjust covariates such as clinical and demographic variables [15]. While MDR is very powerful in detecting the gene-gene interactions for the datasets with a small number of SNPs, however, it is believed that MDR is inefficient in handling large scale GWA data because MDR employs exhaustive searching strategy.

Alternatively, Bayesian and regularization approaches have been proposed for gene-gene interaction analysis such as Bayesian epistasis association mapping (BEAM) [20] and penalized logistic regression models [21]. BEAM is a Bayesian marker partition model to select an optimal marker partition with the highest posterior probability via a Markov Chain Monte Carlo method [20]. Although BEAM was proposed for detecting gene-gene interaction in large scale genetic data, it is not easy to handle high-dimensional data with more than 500,000 SNPS due to its computational complexity [6]. Park and Hastie [21] proposed a stepwise penalized logistic regression (stepPLR) method for detecting gene-gene interactions. In stepPLR, *L_2 _*penalization is utilized, because it provides stable parameter estimates as the dimensionality increases, even if the number of variables is greater than the sample size. Although stepPLR adopted forward selection and penalization to choose the causal SNPs, it suffers from a heavy computational burden when estimating parameters.

Note that all these previous methods for gene-gene interaction analysis are SNP level approaches. That is, gene-gene interaction analysis is performed in SNP level by focusing on SNP-SNP interaction. Unfortunately, these SNP level analyses are not appropriate for handling 500 K to one million SNPs available in GWA studies, because performing gene-gene interaction analysis in SNP level requires huge search spaces and suffers from heavy computational burdens. In the current large scale genome-wide framework, high-order interaction analysis for the SNPs is thus practically impossible.

To overcome such practical issues, two stage procedures have been proposed, in which SNPs that meet some threshold in a test at the first stage analysis are subsequently followed up for modeling interactions at the second stage [22-24]. Although this approach is computationally feasible, there are high possibilities of losing genuine interactions occurring in the absence of marginal SNP effects.

In this paper, we propose a novel gene-based gene-gene interaction method for GWA studies based on MDR analysis scheme. We call our proposed method Gene-MDR analysis. In order to find interacting genetic factors in GWA studies, our Gene-MDR method is composed of two-step applications of MDR analysis: within- and between-gene MDR analyses. Within-gene MDR analysis summarizes each gene's effect from multiple SNPs within the same gene. Between-gene MDR analysis performs the interaction analysis using the summarized gene effects derived from the within-gene MDR analysis step. Furthermore, Gene-MDR has some additional features for the GWA studies. For example, Gene-MDR provides multiple susceptible gene-gene combinations, while other MDR methods report only one combination as the best one.

The proposed Gene-MDR analysis is applied to the GWA study of bipolar disorder (BD) from Wellcome Trust Case Control Consortium (WTCCC). BD is a psychiatric disorder characterized by extreme mood changes experiencing alternating episodes of depression and mania interspersed with periods of normal function [25]. BD is chronic, severely disabling, and life-threatening, with increased risk of suicide and estimated lifetime prevalence of ~1% [25]. In family studies, monozygotic twin concordance rate estimates ranged from 45 to 70% and sibling recurrence risk estimates from 5 to 10 [26]. While this implies strong genetic inheritance, the identification of specific genetic factors related with BD has been difficult [25,27]. Numerous linkage and candidate gene studies have investigated BD linked regions and associated genes, but their results showed highly divergent and inconsistent results, which is due to genetic heterogeneity and substantial polygenic components on BD [25-27]. Recently, several groups have conducted GWA studies and reported genetic factors associated with BD using a conventional single SNP association tests and meta analysis [2,25,28-30].

In this study, in order to understand genetic architecture and identify polygenic components on BD we investigated the gene-gene interaction via Gene-MDR. Application of Gene-MDR to BD GWA data identified several novel high order gene-gene interaction results which cannot be detected by the previous methods.

## Methods

### WTCCC bipolar disorder data

We applied our proposed Gene-MDR method to genome-wide data from the WTCCC, which was the first successful large comprehensive GWA study which included seven complex diseases: BD, cardiovascular disease, hypertension, rheumatoid arthritis, Crohn's disease, type 1 diabetes, and type 2 diabetes, with 2,000 cases for each of the diseases and 3,000 shared common controls [2]. The majority of subjects were of European ancestry. All the individuals were genotyped using Affymetrix GeneChip 500 K arrays. We used the genotype data called by the algorithm CHIAMO for BD and the shared controls, which consisted of the 1958 Birth Cohort (58C) and UK Blood Service sample (NBS) from the WTCCC website.

Prior to analysis, quality control (QC) processes were conducted as follows. (1) Hardy-Weinberg Equilibrium test *P*-value < 5.7 ×10^-7 ^in controls; (2) allelic and/or genotypic association test *P*-value < 5.7 ×10^-7 ^between 58C and NBS; (3) SNPs with minor allele frequency (MAF) < 5% and missing genotype proportion > 5%. Additionally, in order to correct population stratification, we further conducted the principal component analysis using SNPs chosen to reduce inter-locus linkage disequilibrium via EIGENSTRAT [31]. Imputation of missing genotypes was also performed via fastPHASE using options -T 10, -K 20, and -C 30 [32]. After QC process, 354,022 SNPs were remained.

Before performing interaction analysis, we tested the single SNP association with adjustments for sex, age, and first two principal components. From the results, three SNPs, rs1048194, rs12050604, and rs9508846, reached genome-wide significance (*P *< 5 × 10^-8^). However, these SNPs have not been reported in any previous studies using WTCCC data. We guess that these SNPs had been removed in the analysis due to the unreported genotype calling errors. Thus, we excluded these SNPs and then conducted the interaction analyses to detect interacting genetic factors with 354,019 SNPs from 4,806 participants (1868 BD and 2938 controls).

### Gene-MDR method

**Figure 1:**
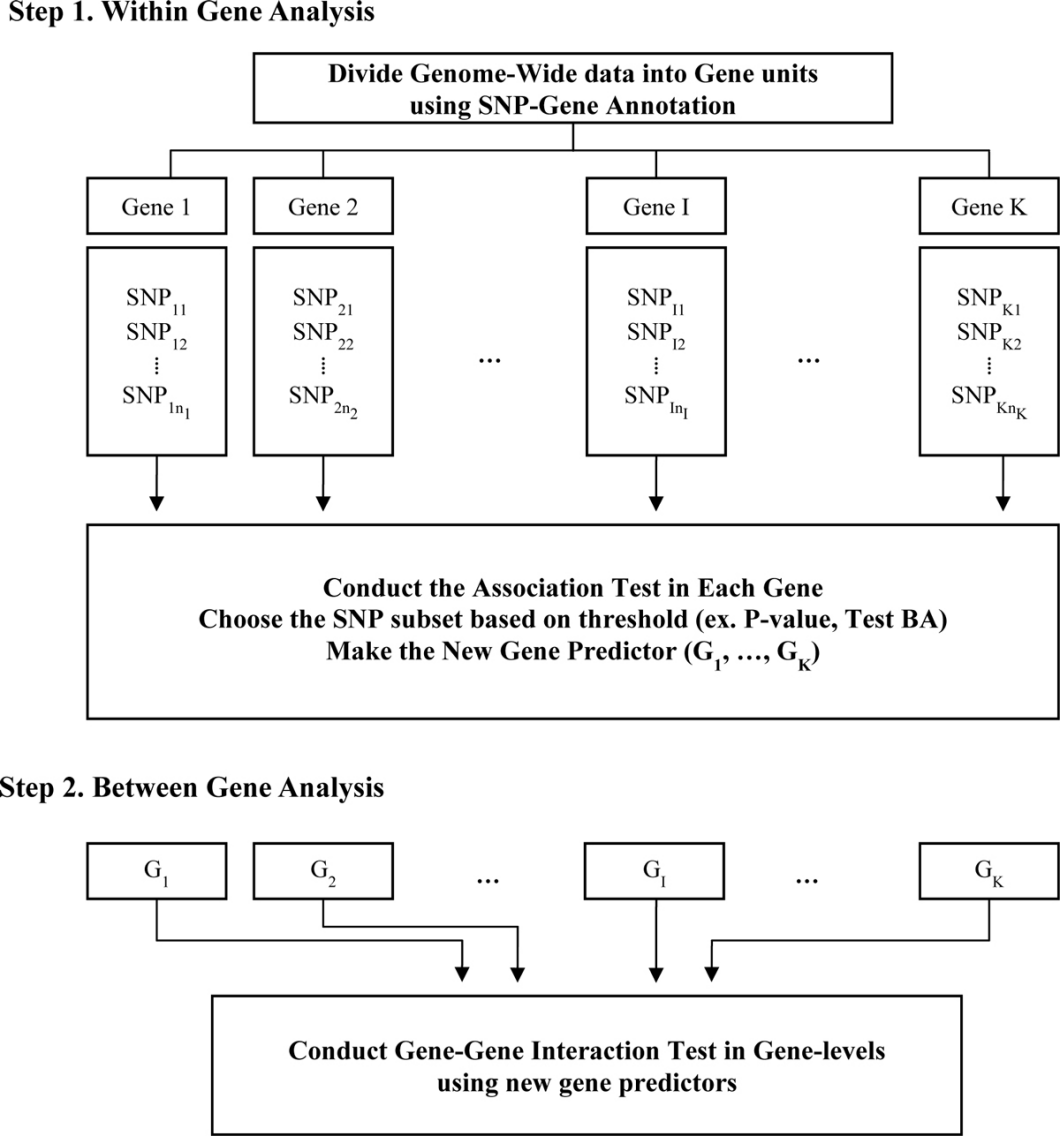
**Flow chart of Gene-MDR analysis**.

The proposed Gene-MDR method is composed of two-step applications of MDR: within- and between-gene MDR analyses. First, within-gene MDR analysis summarizes each gene effect via MDR analysis by combining multiple SNPs from the same gene. Second, between-gene MDR analysis then performs the interaction analysis using the summarized gene effects from within-gene MDR analysis. We describe our proposed method via generalized MDR method. Figure 1 summarizes the detailed procedure of Gene-MDR analysis.

#### Within-gene MDR analysis

The within-gene MDR analysis step is for SNP level analysis. Prior to this analysis, all SNPs in the GWA dataset are allocated to the nearest gene on a basis of annotation information. MDR analysis is then performed for each gene and provides the summarized gene effects in accordance with the best SNP combination.

Since MDR is an exhaustive searching strategy, all the SNP combinations are evaluated for their ability to classify the disease status in the training dataset based on 10-fold CV. For choosing the best SNP combination for each gene, two selection criteria are used. One is the cross validation consistency (CVC) defined as the number of times a particular SNP combination is identified across the 10-fold CV. The other is the average of test balanced accuracy (BA), which is the measure of average of sensitivity and specificity. Therefore, the selected best SNP combination has the highest CVC in each gene. If there are several SNP combinations with the same CVC, SNP combination with a higher test BA value is selected as the best SNP combination. Consequently, the chosen SNP combination has the highest CVC and/or test BA for each gene. For the chosen SNP combination, MDR method classifies each level of SNP combination into the binary class of high/low risk. We call this binary classifier the gene predictor. This gene predictor summarizes the individual gene effect for each gene.

#### Between-gene MDR analysis

The second step is the between-gene MDR analysis step. In this step, the gene predictors with CVC and test BA smaller than the threshold value are excluded because these genes tend to have a low chance of being strongly associated with the disease. MDR analysis for the gene predictors is then performed. The best gene-level combinations are selected using 10-fold CV, similarly as in within-gene MDR analysis.

## Results

### Results of within-gene MDR analysis

Based on the annotation information from Affymetrix, all SNPs were assigned to the nearest gene within 100 kb. If there are no genes within this range, the SNPs were treated as non-annotated and were excluded from the analysis. Finally, 234,748 SNPs located in 17,359 genes on autosomal chromosomes were used in our analysis. Each gene has the different number of SNPs from one to 779 SNPs.

Firstly, we conducted within-gene MDR analysis using generalized MDR in order to adjust for the covariate effects of sex, age, and first two principal components. For simplicity, we considered the interaction up to the fourth order. We selected the best SNP combination in each gene through 10-fold CV using CVC and test BA.

**Figure 2:**
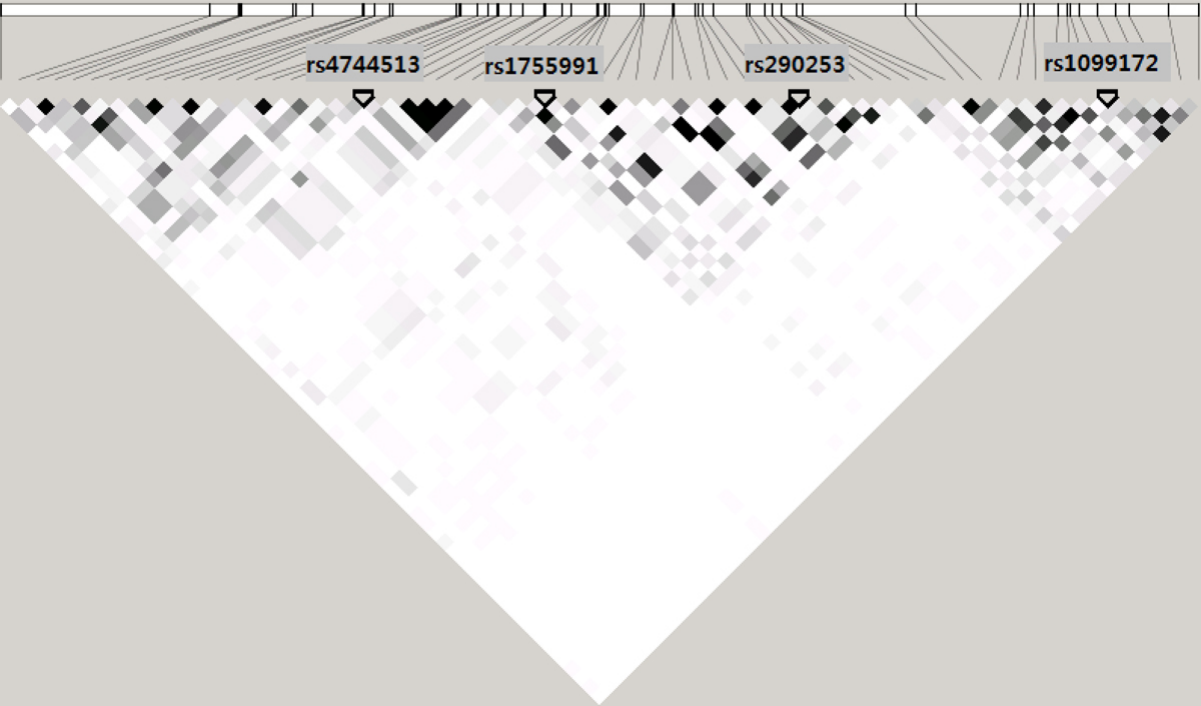
**The LD block of SYK gene**.

Table 1 shows the results of top 10 genes based on test BA in the within-gene MDR analysis step. For example, the top ranked gene is spleen tyrosine kinase (*SYK*) gene in which 67 SNPs were annotated. Among 67 SNPs, the combination with four SNPs was selected as the best SNP combination with the highest value of test BA. It is remarkable that the selected SNPs are not in LD, even though they are in the same gene (Figure 2). All these best combinations showed the similar test BAs (> 0.56). This within-gene MDR analysis step provided the best predictor for each gene.

**Table 1:** Top 10 gene predictors from the within-gene MDR analysis step

Gene	# of SNPs	Genotype combinations	CVC	Test BA
*SYK*	67	rs4744513, rs1755991, rs290253, rs10991725	8	0.5680
*KATNAL1*	40	rs586392, rs641545, rs4572240, rs9551866	6	0.5679
*NIBP*	96	rs10875446, rs6578061, rs7387053, rs11779587	8	0.5677
*ERG*	78	rs1537105, rs2836480, rs743446, rs2836631	9	0.5662
*ZNF385B*	102	rs2138879, rs868273, rs194674, rs260059	9	0.5657
*ZADH2*	25	rs679230, rs9958993, rs1132845, rs2639982	10	0.5652
*NEBL*	101	rs4748697, rs10764274, rs788992, rs631361	9	0.5643
*SYT6*	53	rs611514, rs12027327, rs148210, rs6671620	9	0.5641
*PTPRS*	16	rs8113371, rs11085118, rs11878779, rs3948683	9	0.5640
*KCNIP1*	101	rs1553532, rs6555904, rs329470, rs4868027	8	0.5637

### Results of between-gene analysis

Next, we performed the between-gene MDR analysis step for the gene-gene interactions by using the gene predictors defined in the within-gene MDR step. Before conducting between-gene MDR analysis, we excluded 979 gene predictors with CVC < 5 and/or test BA < 0.5, which had a low chance of being associated with BD. Finally, we performed the MDR analysis for the 16,380 gene predictors via generalized MDR in order to adjust for the covariates in the similar manner as in the within-gene MDR analysis step. We performed all possible two-way interactions and selected the best gene predictor combinations based on the 10-fold CV. Table 2 shows the top 10 two-way gene predictor combinations. The test BA values of all gene combinations were similar.

**Table 2:** Top 10 two-way gene predctor interaction results of the between-gene MDR analysis step and SNP level interaction results

Rank	Gene combinations	Number of SNPs	Test BA	SNP level	
				
				Interaction order	Test BA
1	*ERG, NEBL*	4, 4	0.5833	8	0.6674
2	*ERG, GABBR2*	4, 4	0.5832	8	0.6558
3	*CHST11, ERG*	1, 4	0.5821	5	0.6004
4	*CHST11, KATNAL1*	1, 4	0.5820	5	0.5910
5	*TIAM2, ERG*	4, 4	0.5812	8	0.6585
6	*COLEC12, STARD13*	4, 4	0.5809	8	0.6532
7	*SHANK2, MUC16*	4, 4	0.5808	8	0.6536
8	*KLHL29, GRIN2B*	4, 4	0.5808	8	0.6483
9	*CHST11, KLHL29*	1, 4	0.5807	5	0.5947
10	*CHST11, NIBP*	1, 4	0.5805	5	0.5948

Gene predictors in Table 2 are composed of five to eight SNPs combinations. Thus, these two-way gene predictor combinations correspond to the much higher order interaction in SNP combinations. Practically, it is hard to identify such higher order SNP interactions in current GWA studies. For instance, to identify the 8th order interaction with 500 K SNPs, _500 K_C_8 _= 9.69 × 10^40 ^SNPs combinations have to be tested. However, our Gene-MDR analysis could identify such high order interactions very fast and efficiently.

### Gene-gene interaction analysis in SNP level

In order to examine how well these gene predictors estimate the BD status, we performed the MDR analysis for SNP levels up to the 8th order SNP interactions. Table 2 also shows the SNP level interaction results for the gene predictor interactions.

As shown in Table 2 the test BAs in the SNP level analysis are relatively higher than those from the gene predictor analysis. The hightest value of the test BA is 0.67, which is pretty high rarely found in the usual MDR analysis. The main reason why these test BAs are higher than those from gene level MDR analyses is that the gene level analysis used gene effects summarized from the chosen SNPs. In fact, dichotomization of MDR analysis could lead loss of information [33,34]. For example, v-ets erythroblastosis virus E26 oncogene homolog (*ERG*) and nebulette (*NEBL*) gene combination has the maximum value of test BA in gene level, 0.5833, while the maximum test BA value in SNP level is 0.6674. Despite some possibility of the loss of information in the within-gene MDR step, however, our Gene-MDR is capable of detecting high order of SNP level interactions associated with common complex traits in GWA studies.

### The network plot of gene-gene interaction

**Figure 3:**
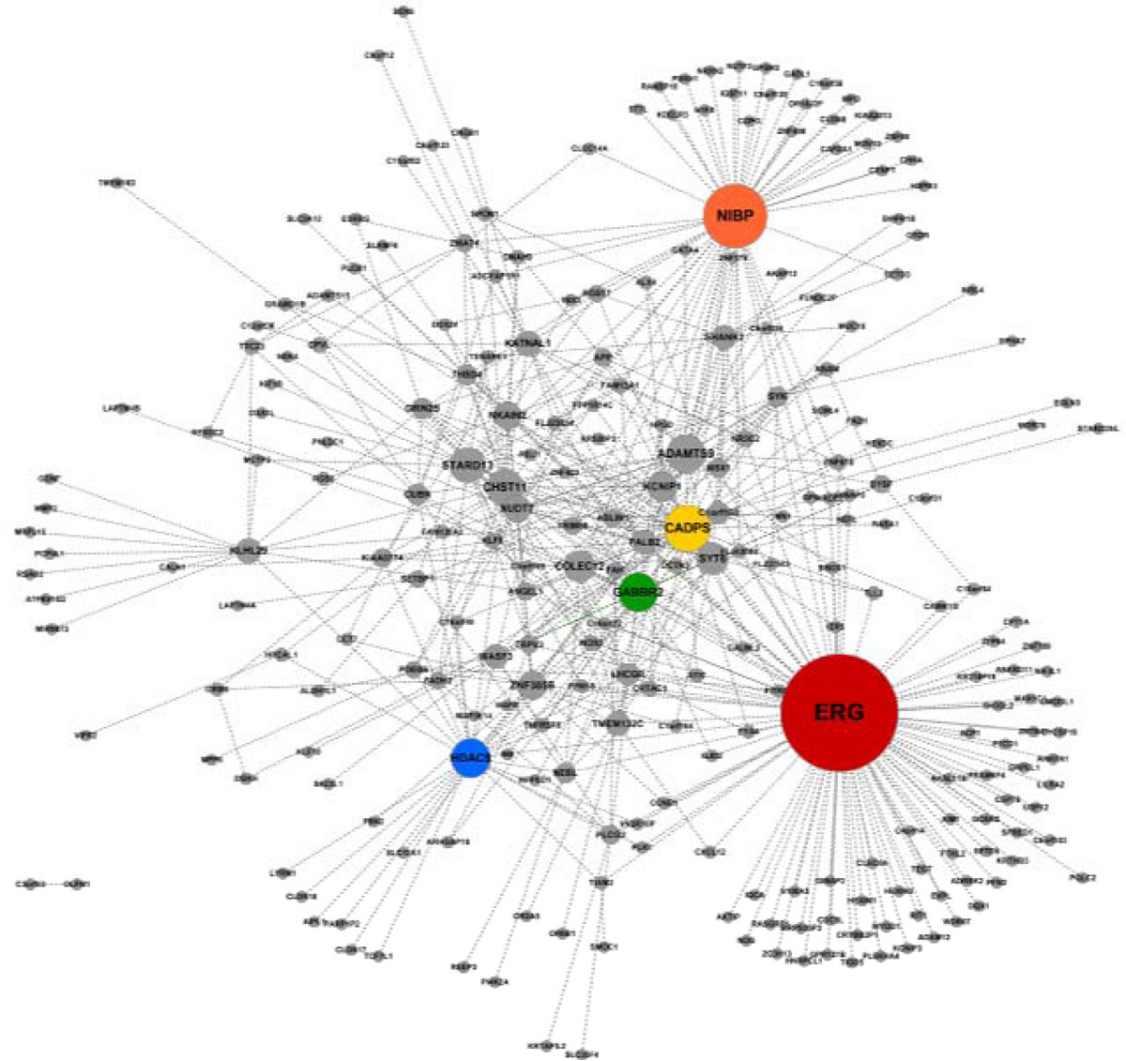
**The network plot of 500 gene-gene interactions**.

Figure 3 shows the network plot of top 500 gene-gene interactions from the BD application result. This network plot shows which gene combinations are highly associated with BD, and which genes play the role of hub genes. In the network plot, a node (point) represents a gene, and a line represents the interaction between two nodes. The size of node represents the degree of interactions with other genes.

We marked five genes with different colors in Figure 3: *ERG*, *NEBL*, Ca^2+^-dependent secretion activator (*CADPS*), gamma-aminobutyric acid (GABA) B receptor, 2 (*GABBR2*), histone deacetylase 9 (*HDAC9*), respectively. These genes could be considered as the hub genes in two-way interactions. For example, among them, the largest node is *ERG *gene, which is interacting with 100 genes. The other genes, *NEBL*, *CADPS*, *GABBR2*, *HDAC9*, are also interacting with 50, 34, 27, and 27 genes, respectively.

## Discussion

We proposed a dimensional reduction analysis for the fast and efficient high order gene-gene interaction analysis via MDR analysis. Our proposed method has the following advantages; (1) it reduces a search space from SNP level to gene level, (2) it is computationally feasible, (3) its results can be directly interpretable in gene level, and (4) its results involve the high-order SNP interactions, which might not be easily identified by other SNP level gene-gene interaction analysis.

Our application of Gene-MDR method to BD data detected many novel susceptible high order gene-gene interactions efficiently. In previous SNP level analyses, *ERG*, *NEBL*, *CADPS2*, *GABBR2*, and *HDAC9 *genes have been reported to be related with neuropsychiatric diseases such as BD, depression disorder, and schizophrenia [35-44]. *ERG *gene is located on 21q22.3, which is one of widely studied regions for BD. Since Straub et al. first reported the evidence of linkage in a large multigenerational pedigree with a maximum lod score of 3.41 with the *PFKL *locus on 21q22.3 [35], several other groups have reported the evidence of linkage for BD and 21q22 [36]. Recently, *ERG *gene was reported to be very close to the markers in positive linkage with BD [37]. *NEBL *gene is located on 10p12, which encodes a nebulin like protein that is abundantly expressed in cardiac muscle. While the relatedness between this gene and cardiomyopathy is reported from many groups, *NEBL *is also associated with depression disorder. In a genome-wide analysis of suicidal thoughts and behavior in major depression from the RADIANT study, minor allele of one variant in *NEBL *gene is reported to be associated with suicide attempt [38].

The remained *CADPS2*, *GABBR2*, and *DHAC9 *genes are related with schizophrenia. In the study of brains of schizophrenia patients, Hattori et al. examined the expression of *CADPS2 *mRNA in the postmortem brains (BA6) of psychiatric patients (schizophrenia, major depression, and BD) and controls. A significant increase in *CADPS2 *expression was detected in the brains of the schizophrenia group, compared to the control group [39]. *GABBR2 *on 9q22.1-q22.3 is a well-known gene, as a susceptibility locus for schizophrenia [40] and another gene in this locus is reported to be associated with cognitive test measure [41]. *HDAC9 *on 7p21.1 is highly expressed in brain and skeletal muscle [42]. Tam et al. reported that *HDAC9 *gene was found to contain single schizophrenia-specific deletions in copy number variation study [43]. A decrease in the expression of this gene has been associated with increased neuronal apoptosis [43,44].

Interestingly, while these five genes were shown to play the role of hub genes in our study, they could not be detected from the current genome-wide approaches, because no SNPs in these genes were marginally associated with BD in genome-wide significance level. All *P*-values were greater than 1 × 10^-4^. However, Gene-MDR method detected such many susceptible and candidate gene-gene interactions efficiently in genome-wide scale.

While several methods have been proposed for identifying gene-gene interaction in GWA studies, all of these methods are SNP level analysis and cannot be practically applied to the GWA data, because huge search spaces and heavy computing are required. Especially, the exhaustive search methods such as the MDR method cannot identify high order gene-gene interaction from GWA data. In order to address these challenges, we propose the Gene-MDR approach which is an efficient and fast gene-based gene-gene interaction analysis method for GWA data. By compromising between an exhaustive search method and the two-stage analysis method, Gene-MDR can easily conduct interaction analysis in gene level and detect high order interactions in SNP level.

Even though Gene-MDR is quite efficient for GWA analysis, it needs some further investigations regarding the following issues. First, Gene-MDR uses SNP-gene annotated information. While Gene-MDR can consider wide mapping region such as between 200 kb and 500 kb, we chose the 100 kb mapping region in order to avoid overlap between two adjacent genes and to have a better interpretation of the gene function by restricting mapping range. As a result, about 1/3 SNPs in WTCCC dataset were not annotated and excluded in the analysis. The loss of genetic information depending on the choice of mapping ranges needs to be investigated for the gene-based approach. Second, Gene-MDR may not provide the globally best gene-gene interactions, because it is based on the summarized gene-level information. Therefore, the genuine epistatic factors could be missed. Third, each gene has a different number of SNPs. Thus, the use of the same fixed number of SNPs may not be optimal. Since in our BD analysis we summarized the gene effects using up to four SNPs, the gene predictors might have insufficient information about SNPs. Although our Gene-MDR can easily handle the higher order interaction greater than four, we think the 8th order interaction in SNP levels would be high enough to represent high order interaction of SNPs.

In this study, we did not compare the performance of Gene-MDR with other commonly used gene-gene interaction methods. Since our Gene-MDR is the gene level approach and others are the SNP level approaches, however, it is difficult to perform a direct comparison. Furthermore, other methods based on the SNP levels cannot handle the 8^th ^order interaction.

## Conclusion

Gene-gene interaction analysis is important in that it can provide the clue to understand the etiology of complex diseases. In this respect, our proposed method has the following advantages in applications to GWA studies. First by reducing high dimensional data from SNPs to gene, Gene-MDR can analyze the gene-gene interaction with a relatively small number of gene predictors in GWA studies. Additionally, Gene-MDR can reduce computation time severely for conducting gene-gene interaction analysis. As a simple example, assume that there are 500 K SNPs from 5,000 samples, and each gene has 10 SNPs. When we conduct gene-gene interaction analysis using 10-fold CV with 2-GHz Dual Core AMD Opteron(tm) processor (8 GB RAM) in Linux system, a computing time of the 2nd order SNP level MDR analysis was 38,749,922.5 seconds. On the other hand, our Gene-MDR took 393,492.2 seconds while considering the 8th order SNP interactions. Hence, Gene-MDR used 1/100 computation time than SNP level MDR analysis.

Second, it is possible to detect high-order interaction using gene predictors. From the results of Gene-MDR, we can trace the high order interaction in SNP level from the results of the between-gene MDR step. As shown in our BD application, when a two-way interaction between gene predictors is identified via our Gene-MDR method, it may correspond to the 5th to 8th SNP order interaction.

Finally, the idea of our method can be applied to other gene-gene interaction approaches. Various statistical methods such as a principal component analysis and a factor analysis can be applied by using the summarized gene predictors. We will investigate the performance of the summarized gene predictors in other gene-gene interaction methods in the near future.

## Competing interests

The authors declare that they have no competing interests.

## Authors' contributions

SO conceived and designed the method, analyzed the data, and drafted the manuscript. JH designed the method. MSK performed the gene annotation. BW and KH critically read the manuscript. TP coordinated the work, conceived and designed the method, drafted the manuscript, and made the major edits. All of the authors read and approved the manuscript.
